# Achalasia with esophageal intramural hematoma treated by per‐oral endoscopic myotomy (POEM)

**DOI:** 10.1002/deo2.70

**Published:** 2021-11-02

**Authors:** Yusuke Fujiyoshi, Mary Raina Angeli Fujiyoshi, Ryusuke Kimura, Hiroki Shinohara, Yohei Nishikawa, Yuto Shimamura, Haruo Ikeda, Manabu Onimaru, Haruhiro Inoue

**Affiliations:** ^1^ Digestive Diseases Center Showa University Koto Toyosu Hospital Tokyo Japan

**Keywords:** achalasia, intramural hematoma, per‐oral endoscopic myotomy, POEM

## Abstract

Esophageal intramural hematoma (EIH) is a condition which occurs as a result of hemorrhage within the esophageal wall including the submucosal layer. However, reports of EIH on achalasia patients are quite limited and per‐oral endoscopic myotomy (POEM) for achalasia with EIH has not been reported. This is the first case report that demonstrated a successful treatment of achalasia with EIH by POEM. In achalasia, since there is absence of lower esophageal sphincter relaxation, as barotraumatic pathogenesis, an increase in the intraesophageal pressure may cause EIH. As direct traumatic pathogenesis, the stasis of food may directly injure the esophageal wall resulting in EIH. After confirming the hematoma healed until it became an ulcer, POEM was performed on the posterior axis since the intramural hematoma was located anteriorly. The procedure was completed successfully without any occurrence of adverse events. On 2‐months follow‐up, improvement in dysphagia was noted, and complete epithelialization of the intramural hematoma region was seen on endoscopic examination. On 1‐year follow‐up, patient did not have recurrence of dysphagia and intramural hematoma. In summary, we reported a case of achalasia with EIH, which was treated by POEM. POEM procedure may be effective not only for the improvement of dysphagia but also for a better ulcer healing and prevention of intramural hematoma recurrence.

## INTRODUCTION

Achalasia is a primary esophageal motility disorder with impaired relaxation of the esophagogastric junction (EGJ), along with the loss of organized peristalsis in the esophageal body.^1^ The typical symptoms are dysphagia for solids and liquids without oropharyngeal transfer difficulties, regurgitation, weight loss, and chest pain. Per‐oral endoscopic myotomy (POEM) is reported to be an effective minimally invasive treatment of achalasia.^2^

Esophageal intramural hematoma (EIH) is a condition which occurs as a result of hemorrhage within the esophageal wall including the submucosal layer.^3^ The underlying etiology or predisposing factors were reported to be esophageal instrumentation, vomiting, trauma, pill‐induced injury, food impaction‐related issue, or coagulation defects.^4^ However, reports of EIH on achalasia patients are quite limited,^5^, ^6^, ^7^, ^8^, ^9^ and POEM procedure for achalasia with EIH has not been reported.

Herein, we report a case of a patient examined at our institution and diagnosed with achalasia with EIH, treated successfully by POEM.

## CASE REPORT

**FIGURE 1:**
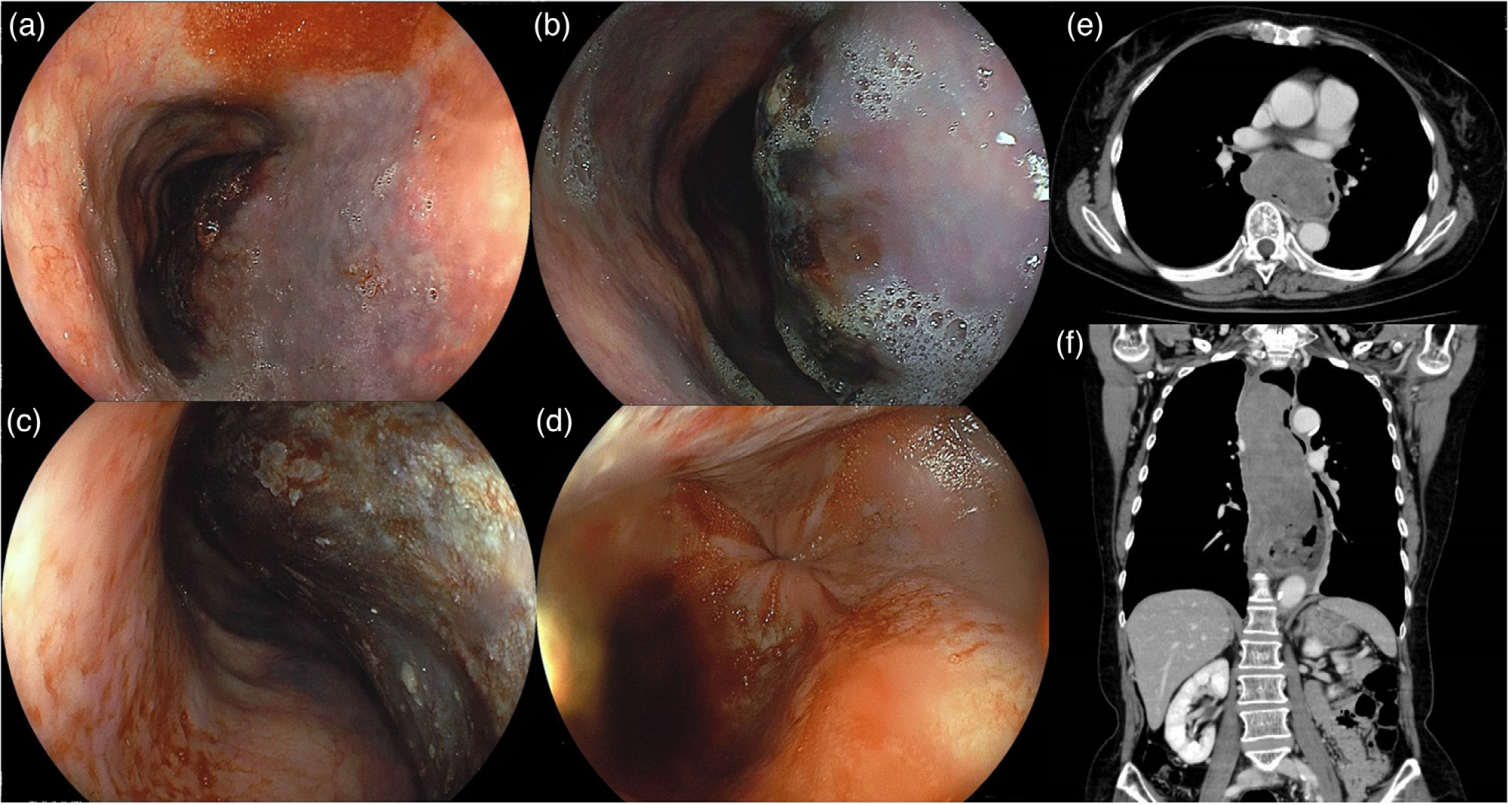
Esophagogastroduodenoscopy (EGD) showed a large intramural hematoma from upper esophagus to lower esophagus on the anterior wall (a‐c). Esophagogastric junction (EGJ) was tight and showed esophageal rosette sign (d). Chest and abdominal CT image of the esophageal intramural hematoma (EIH), which is a slight hyperdense mass in the dilated esophagus (e and f)

This is a case of a 68‐year‐old female who presented with a 40‐year history of mild, occasional dysphagia, and chest pain. Previous consultation was done at a different institution 40 years ago wherein an upper endoscopy was performed, and achalasia was suspected. However, since there were no accompanying symptoms such as nausea, vomiting, or unintentional weight loss, patient was followed‐up without any treatments. She has no significant past medical history and medications. Due to a recent sudden onset of melena and dysphagia, she was admitted at a previous institution. Laboratory results revealed normocytic anemia (hemoglobin level of 8.1g/dl). CT scan of the thorax and upper abdomen revealed a slight hyperdense mass in the dilated esophagus, which was suspected to be EIH (Figure 1e,f). Esophagogastroduodenoscopy (EGD) showed a large intramural hematoma in esophageal body (20‐36cm from the incisors) (Figure 1a‐c). The EGJ was tight and showed esophageal rosette sign (Figure 1d). She was managed as nil by mouth and was subsequently referred to us for further management 7 days after the onset.

**FIGURE 2:**
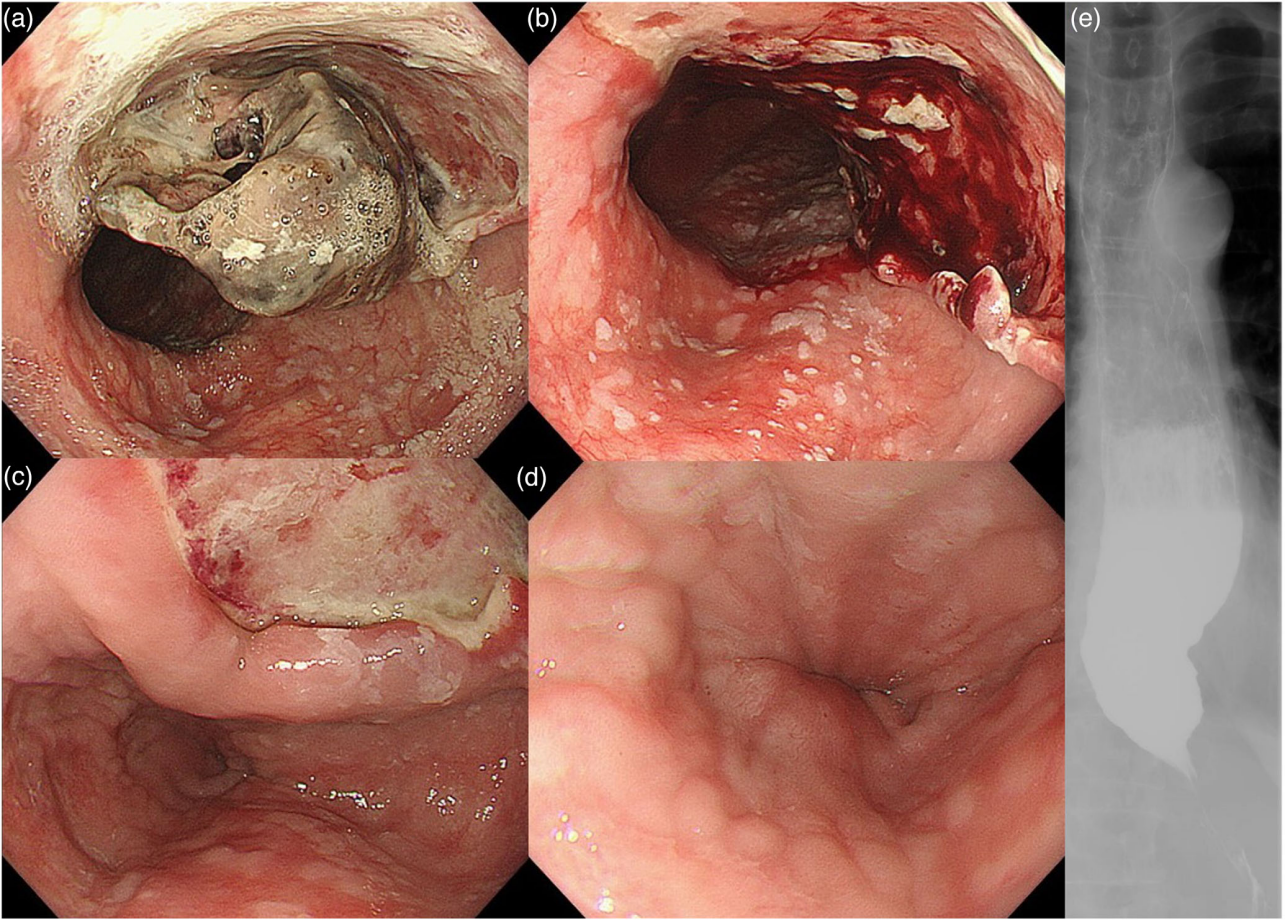
Follow‐up esophagogastroduodenoscopy (EGD) showed a dissected necrotic mass lesion with submucosal ulceration (a). The necrotic mass easily came off and showed submucosal ulceration, which was located on the same part of the intramural hematoma (b and c). Esophagogastric junction (EGJ) was tight and showed esophageal rosette sign (d). Barium esophagram showed dilated esophageal body, stasis of barium, and constricted EGJ (e)

The patient underwent evaluation for achalasia which includes EGD, barium esophagram, and high‐resolution manometry (HRM). The EGD showed a dissected necrotic mass lesion with submucosal ulceration (Figure 2a). The necrotic mass easily came off and showed submucosal ulceration, which was in the same part of the intramural hematoma (Figure 2b,c) indicating that the EIH healed conservatively, and it became an ulcer. The EGJ, which was 42 cm from the incisors, was tight and showed esophageal rosette sign (Figure 2d). HRM showed a high integrated relaxation pressure (23.3 mm Hg) and lower esophageal sphincter (LES) pressure (37.0 mm Hg), and absence of peristalsis in esophageal body. Barium esophagram showed a dilated esophageal body, stasis of barium and constricted EGJ (Figure 2e). These findings were consistent with the diagnosis of Type I achalasia on Chicago classification version 4.0. After the discussion with the patient, POEM was decided to be performed. This procedure has been approved by the ethics committee of Showa University Koto Toyosu Hospital and was performed in accordance with the Declaration of Helsinki. Written informed consent was obtained from the patient prior to the procedure.

**FIGURE 3:**
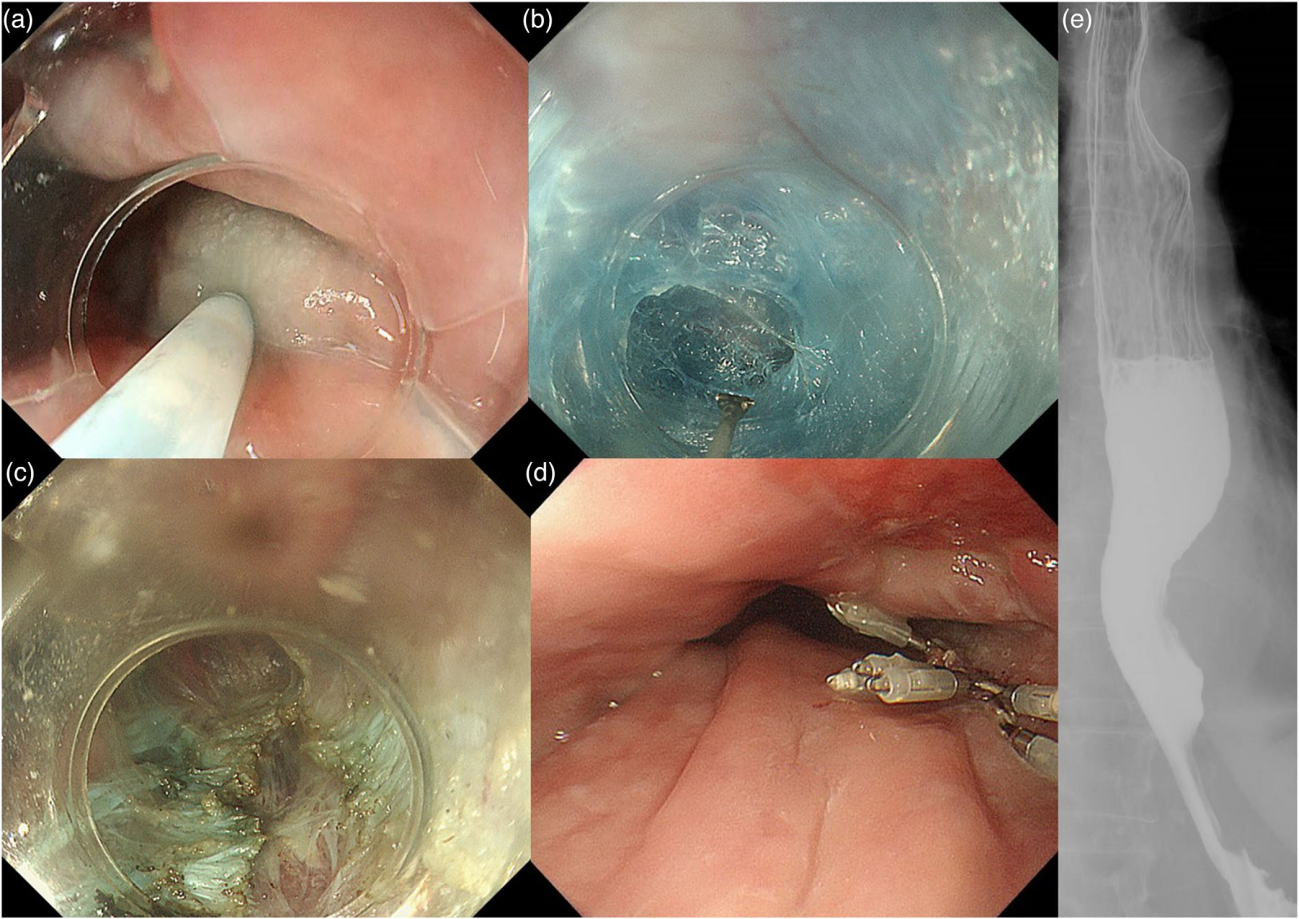
Esophagogastroduodenoscopy (EGD) images of per‐oral endoscopic myotomy (POEM) procedure. Submucosal injection was done at the posterior wall (a). Submucosal tunnel was created until the gastric side (b). Myotomy of internal circular muscle was done along the submucosal tunnel preserving the longitudinal muscle (c). Entry site was completely closed with endoclips (d). Barium esophagram 1 day after POEM showed the opening of the esophagogastric junction (EGJ) (e)

**FIGURE 4:**
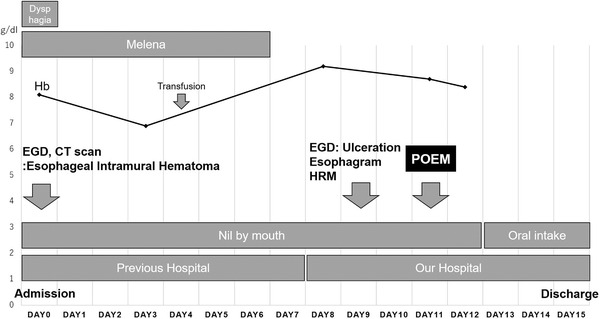
Patient's clinical course from the onset of the esophageal intramural hematoma

Since the ulcer was located on the anterior wall of the esophagus (11–3 o'clock axis, 20–36 cm from the incisors), the submucosal injection was done, and the entry site was created on the posterior wall (4 o'clock axis, 36 cm from the incisors) (Figure 3a). Following this, a submucosal tunnel was created until the gastric side, going to the lesser curvature of the stomach (Figure 3b). During the procedure, no fibrosis in the submucosal space was seen. To confirm the appropriateness of the length of the submucosal tunnel, the double scope method was performed wherein the main scope was inserted into the submucosal tunnel, and an ultra‐thin endoscope was inserted into the stomach. With the ultra‐thin endoscope on retroflexion, the submucosal tunnel length was confirmed by the illumination of the main scope. Thereafter, selective myotomy of the internal circular muscle was done along the submucosal tunnel while preserving the longitudinal muscle (Figure 3c). The entry site was then completely closed with endoclips (Figure 3d). The procedure was successfully completed without any adverse events. No complications on second‐look endoscopy were noted, and adequate opening of the EGJ was seen on barium esophagram (Figure 3e). Oral intake was started on postoperation day (POD) 2, and the dysphagia was noted to be resolved. Patient was then discharged on POD 4. On 2‐months follow up, the improvement of dysphagia was noted and complete epithelialization of the intramural hematoma region was seen on EGD. On 1‐year follow‐up, patient had no recurrence of dysphagia or intramural hematoma. Patient's clinical course from the onset of the EIH is presented in Figure 4.

## DISCUSSION

In this case report, we presented a case of achalasia with EIH, which was treated by POEM for dysphagia management and prevention of EIH recurrence. To our knowledge, only five cases of achalasia with EIH have been reported.^5^, ^6^, ^7^, ^8^, ^9^ Of the five cases previously reported, two were treated with Heller's myotomy, one with surgery, one with endoscopic dilatation, and one with medication. One case treated with Heller's myotomy showed recurrence of EIH after 15 months,^5^ and one with medication showed recurrence of EIH after 7 years.^8^ Our study is the first to report on POEM for achalasia with EIH.

Regarding the pathogenesis of EIH, two forms of trauma have been described, barotrauma and direct trauma. Esophageal barotrauma occurs as a result of changes in acute, transmural pressure associated with sneezing, retching or lifting heavy weights.^3^, ^10^ Direct trauma results from endoscopic esophageal manipulation or a large, swallowed food bolus.^3^, ^10^ Aside from these, as the pathogenesis and the nature of the hemorrhage of EIH, coagulation disorders, related to aortic disease, and idiopathic have been reported.^3^ In achalasia, since there is absence of LES relaxation, an increase in the intraesophageal pressure is observed. As barotraumatic factor, with the continuous rise in the intramural pressure, subsequent rupture through the mucosa or the muscularis results in hemorrhage, causing intramural hematoma formation.^3^ In addition, the extension of esophageal body by stasis of food may cause the extension of submucosal layer, contributing to the intramural hemorrhage. Related to direct traumatic pathogenesis, in achalasia, one contributing factor is the stasis of food which may directly injure the esophageal wall resulting in the intramural hemorrhage. In the case of our patient, no coagulation disorders and aortic disease were noted.

In this case, the hematoma healed by conservative treatment until it became an ulcer. In terms of esophageal ulcer healing, there is no effective medication similar to proton pump inhibitors for gastric ulcers or gastroesophageal reflux disease; therefore, freeing the food outlet obstruction was thought to be the only thing that we can theoretically do for promoting the healing of the ulcer. By performing POEM, the food is less likely to stagnate in the esophagus which may contribute positively on ulcer healing. Since the ulcer was located 20–36 cm from incisors, at 11–3 o'clock axis, and was not in the lower esophagus, we were able to create the submucosal tunnel (4 o'clock axis, 36 cm from the incisors) and perform myotomy, avoiding going over the ulcer. Accordingly, we thought we did not have to wait for the complete ulcer healing to perform POEM. In addition, considering that there have been some reports on the recurrence of EIH on achalasia,^5^, ^6^ POEM may also contribute in preventing the recurrence of the intramural hematoma.

In summary, we reported a case of achalasia with EIH, which was treated by POEM. POEM procedure may be effective not only for the improvement of dysphagia but also for a better ulcer healing and prevention of intramural hematoma recurrence.

## CONFLICT OF INTERESTS

Inoue H is an advisor of Olympus Corporation and Top Corporation. He has also received educational grants from Olympus Corp., and Takeda Pharmaceutical Co. Fujiyoshi Y, Fujiyoshi MRA, Kimura R, Shinohara H, Nishikawa Y, Shimamura Y, Ikeda H, and Onimaru M have no conflict of interests to declare.

## FUNDING INFORMATION

None.
